# A review of recent advances in the spherical harmonics expansion method for semiconductor device simulation

**DOI:** 10.1007/s10825-016-0828-z

**Published:** 2016-05-11

**Authors:** K. Rupp, C. Jungemann, S.-M. Hong, M. Bina, T. Grasser, A. Jüngel

**Affiliations:** 1 Institute for Microelectronics, TU Wien, Gusshausstrasse 27-29/E360, 1040 Wien, Austria; 2 Institut für Theoretische Elektrotechnik, RWTH Aachen, Kackertstraße 15-17, 52072 Aachen, Germany; 3 School of Information and Communications, Gwangju Institute of Science and Technology, Gwangju, 500-712 South Korea; 4 Institute for Analysis and Scientific Computing, TU Wien, Wiedner Hauptstrasse 8-10/E101, 1040 Wien, Austria

**Keywords:** Spherical harmonics, Boltzmann transport equation, Semiconductor device simulation, Silicon, Germanium, Hot carrier degradation, Box integration

## Abstract

The Boltzmann transport equation is commonly considered to be the best semi-classical description of carrier transport in semiconductors, providing precise information about the distribution of carriers with respect to time (one dimension), location (three dimensions), and momentum (three dimensions). However, numerical solutions for the seven-dimensional carrier distribution functions are very demanding. The most common solution approach is the stochastic Monte Carlo method, because the gigabytes of memory requirements of deterministic direct solution approaches has not been available until recently. As a remedy, the higher accuracy provided by solutions of the Boltzmann transport equation is often exchanged for lower computational expense by using simpler models based on macroscopic quantities such as carrier density and mean carrier velocity. Recent developments for the deterministic spherical harmonics expansion method have reduced the computational cost for solving the Boltzmann transport equation, enabling the computation of carrier distribution functions even for spatially three-dimensional device simulations within minutes to hours. We summarize recent progress for the spherical harmonics expansion method and show that small currents, reasonable execution times, and rare events such as low-frequency noise, which are all hard or even impossible to simulate with the established Monte Carlo method, can be handled in a straight-forward manner. The applicability of the method for important practical applications is demonstrated for noise simulation, small-signal analysis, hot-carrier degradation, and avalanche breakdown.

## Introduction

Moment-based approaches for semiconductor device simulations are, despite their deficiencies for scaled-down devices, still the most popular methods for technology computer-aided design (TCAD). For example, the drift-diffusion and hydrodynamic models are still used to predict the device characteristics of scaled-down devices, even though their limitations and deficiencies in this regime are well known [1]. These deficiencies could not be addressed through models obtained by taking higher moments either, as closure conditions are hard to formulate and need to rely on empirical arguments [2].

Higher accuracy than that provided by moment-based methods can in principle be obtained by solving the full Boltzmann transport equation (BTE) for the carrier probability distribution function $f(\varvec{x}, \varvec{p}, t)$, where $\varvec{x}$ denotes the spatial coordinate, $\varvec{p}$ momentum, and *t* time. While moment-based models only provide information about averaged quantities such as mean velocity of the particle ensemble centered at a given spatial location $\varvec{x}$, solutions of the BTE provide full information about the distribution of carriers with respect to their momentum. This allows for a full consideration of many details such as scattering processes and high-energy effects. On the other hand, the additional momentum coordinates require a numerical resolution of the momentum space at each spatial discretization element in one way or another. Therefore, the computational effort for solving the BTE is considerably higher than for moment-based models.

The Monte Carlo method was one of the first methods used to solve the BTE for semiconductors and is still the most popular method used today. It provides several appealing advantages for practical use: First, implementations are relatively easy, hence first results can be obtained quickly. Second, many complicated physical details such as sophisticated bandstructures can be included with the Monte Carlo method. Third, the Monte Carlo method is fairly robust because it does not involve the solution of large systems of nonlinearly coupled equation, where divergence may occur, but instead relies on stochastic sampling. On the other hand, the stochastic nature of the Monte Carlo method is also responsible for major shortcomings. The first is due to the inversely proportional relationship of the accuracy with the square root of the number of particles and thus also processor cycles [3]: If the distribution function needs to be resolved over several orders of magnitude, excessive execution times are required [4]. This is the case if rare events or small currents need to be resolved, or if small-signal analysis is performed. Also, the square-root dependence mandates that scaling Monte Carlo simulations beyond the computational resources power provided by a single workstation or a single cluster yields diminishing returns when considering the additional resources invested. The second shortcoming of the Monte Carlo method is the inherent transient nature: Self-consistent device simulations require time steps on the order of femtoseconds to resolve plasma oscillations, hence simulations of time intervals in the millisecond regime or beyond become practically infeasible [5].

To overcome the limited accuracy of moment-based methods on the one hand, but to avoid excessive execution times of the Monte Carlo method on the other hand, sophisticated deterministic methods for solving the BTE were developed. A full discretization of the $(\varvec{x}, \varvec{p})$-space for solving the transient BTE using a weighted essentially non-oscillatory (WENO) scheme for stabilization was proposed by Carillo et al. for one-dimensional simulations [6] and later extended to two-dimensional device simulations (cf. [7] and references therein). Spherical coordinates were used in momentum space in order to better resolve the spherical symmetry of the analytical band structures employed [8, 9]. A related method was proposed by Galler et al. [10], where the multigroup equations are solved using a finite element-like decomposition of the full momentum space into tiny cells.

The most mature deterministic method so far and the focus of this review is the spherical harmonics expansion (SHE) method. It has been successfully employed for a much wider range of device quantities than any of the other direct solution approaches. The SHE method mathematically exploits the fact that the distribution of carrier momentum in equilibrium shows a spherical symmetry. As a consequence, the equilibrium distribution function can be represented exactly with a zeroth-order expansion. This is in contrast to moment-based methods, where higher-order moments do not vanish in equilibrium. Since expansions in spherical harmonics can be seen as an extension of Fourier series from the circle (i.e., one angular component $\varphi$) to the sphere (i.e., two angular components $\theta$, $\varphi$), a rich mathematical foundation is available [11–13].

First numerical applications of the SHE method for solving the BTE for homogeneous semiconductors can already be found in the 1960s, for example, in the work of Baraff [14]. However, it took until the early 1990s until the SHE method was first used for device simulation [15–17]. In these early works, the SHE method was derived from a perturbation of the equilibrium state, resulting in a first-order SHE method for which promising agreement with Monte Carlo results was obtained. Subsequently, several authors refined the method: Lin et al. derived a Scharfetter-Gummel-type stabilization for the first-order SHE method [18], and later coupled it with the Poisson equation and a hole continuity equation [19]. Hennacy et al. extended the SHE method to arbitrary order [20, 21]. Schroeder et al. proposed physically sound boundary conditions [22] to address sharp boundary layers when forcing the distribution function to equilibrium at the contacts. Vecchi et al. introduced a methodology to include full-band effects [23, 24]. The same group proposed an efficient solution scheme with a multigrid-like refinement near the conduction band edge and observed a decoupling of the system of linear equations after discretization when using certain scattering processes [25]. Singh identified a regularizing behavior of inelastic scattering mechanisms over elastic ones and used this to construct a positivity-preserving discretization [26]. Rahmat et al. were the first to observe a decoupling of spatial and angular terms and presented results for a third-order discretization using an upwind scheme [27]. The Bologna group proposed a methodology for handling impact ionization [28], hot electron injections [29], and electron-electron scattering [30, 31] for first-order SHE. In order to consider certain quantum mechanical effects, Goldsman  et al. applied a first-order SHE to a modified BTE taking local contributions from the Wigner equation into account [32]. At about the same time, Ben Abdallah [33, 34] and Ringhofer [35–38] provided important foundations for a better mathematical understanding of the BTE in general and the SHE method in particular. This understanding was supplemented by the results of Hansen  et al., who used the SHE method for the modeling of plasma physics [39].

In the following, we provide a unified presentation of recent improvements of the SHE method. We focus on contributions since the work of Jungemann et al. in 2006 [40], who introduced a sound mathematical stabilization and demonstrated the need for higher-order expansions for nanoscale devices. Section 2 introduces the SHE method at the continuous level with some details on the choice of boundary conditions. The inclusion of additional physical processes and application scenarios of the SHE method are presented in Sect. 3. Section 4 discusses the current state-of-the-art discretization and summarizes various techniques developed for further reducing overall simulation times. Rather than providing a separate results section, we present results directly at the respective point of discussion to preserve a coherent flow of discussion. Finally, we draw a conclusion and discuss possible future research directions worthwhile to pursue.

## The SHE method

In semi-classical transport theory, the spatial and temporal evolution of particles is described by a distribution function $f(\varvec{x}, \varvec{p}, t)$. In the following, we use the quantum mechanical relation $\varvec{p} = \hbar \varvec{k}^\prime$ with reduced Planck constant $\hbar$ in order to switch to the wave vector $\varvec{k}^\prime$. The distribution function then obeys the BTE

1 $$\begin{aligned} \frac{\partial f}{\partial t} + \varvec{v} \cdot \nabla _{\varvec{x}} f + \frac{1}{\hbar }\varvec{F} \cdot \nabla _{\varvec{k}^\prime } f = Q\{ f \} , \end{aligned}$$

where $\varvec{v}$ denotes the group velocity, $\varvec{F} = - \nabla _{\varvec{x}}(\mathrm {q} \psi + \varepsilon _{\mathrm b})$ is the force due to the particle charge $\mathrm {q}$ (negative for electrons, positive for holes), $\psi$ is the electrostatic potential obtained either through self-consistent solutions with the Poisson equation or externally prescribed (frozen field), $\varepsilon _{\mathrm b}$ identifies the band edge (minimum for electrons, maximum for holes), and *Q* refers to the scattering operator. The force term may also depend on the magnetic field [41], which will be neglected in this work for the sake of conciseness. In principle, a BTE needs to be solved for each valley and each carrier type. Interactions between the different valleys and carrier types occur through intervalley scattering and generation-recombination processes. For better readability, the subsequent discussion assumes a single valley for a single carrier type unless noted otherwise and arguments are suppressed whenever appropriate.

### Spherical harmonics expansion

The SHE method allows for accounting for spherical symmetries with respect to momentum. However, common descriptions of the bandstructure in silicon show elliptical symmetries. The elliptical symmetries in the original $\varvec{k}^\prime$ coordinates are mapped onto spherical symmetries using the Herring–Vogt transformation [42]

$$\begin{aligned} \hat{T} = \left( \begin{array}{c@{\quad }c@{\quad }c} T_x &{} 0 &{} 0 \\ 0 &{} T_y &{} 0 \\ 0 &{} 0 &{} T_z \end{array} \right) \end{aligned}$$

via $\varvec{k} = \hat{T} \varvec{k}^\prime$. The BTE (1) after the Herring–Vogt transformation takes the form

2 $$\begin{aligned} \frac{\partial f}{\partial t} + \hat{T} \varvec{v} \cdot \nabla _{\varvec{x}} f + \frac{1}{\hbar } \hat{T}\varvec{F} \cdot \nabla _{\varvec{k}} f = Q\{ f \}. \end{aligned}$$

A SHE can in principle be carried out for constant modulus $k = \Vert \varvec{k} \Vert$ of the transformed wave vector, or for constant kinetic energy $\varepsilon$. An expansion with respect to energy has several advantages: For example, the distribution function is isotropic on equienergy surfaces in equilibrium and many scattering rates are in good approximation independent of the angles [40]. Thus, spherical coordinates $(k, \theta , \varphi )$ in $\varvec{k}$-space are mapped to spherical coordinates $(\varepsilon , \theta , \varphi )$ in energy space, where we keep the angles unchanged and require the mapping to be unique in both directions [43]. Such a one-to-one mapping is naturally fulfilled for the analytical Modena model, which will be discussed in Sect. 3.1. Moreover, as we will later see in Sect. 3.1, the requirement of a one-to-one mapping can be relaxed substantially.

A SHE of an arbitrary function *u* in energy space reads

3 $$\begin{aligned} u(\varvec{x}, \varvec{k}(\varepsilon , \theta , \varphi ), t) = \sum _{l=0}^\infty \sum _{m=-l}^l u_{l,m}(\varvec{x}, \varepsilon , t) Y^{l,m}(\theta , \varphi ) , \end{aligned}$$

where $Y^{l,m}$ are the orthonormal, real-valued spherical harmonics on the unit sphere. Conversely, for any given function *u* on the unit sphere with mild regularity requirements, the expansion coefficients $u_{l,m}$ are obtained from a projection onto the respective spherical harmonic [11]:

4 $$\begin{aligned} u_{l,m} = \int _{\partial \varOmega } u Y^{l,m} \, \mathrm {d}\varOmega \end{aligned}$$

Here, $\varOmega$ denotes the unit sphere and $\, \mathrm {d}\varOmega = \sin \theta \, \mathrm {d}\theta \, \mathrm {d}\varphi$. The description of the BTE in $\varvec{k}$-space requires a projection of a function *u* over the whole Brillouin zone $\mathcal {B}$ for a kinetic energy $\varepsilon$ as

5 $$\begin{aligned} \frac{1}{(2\pi )^3} \int _{\mathcal {B}} \delta (\varepsilon - \varepsilon (\varvec{k})) Y^{l,m} u \, \mathrm {d}\varvec{k} , \end{aligned}$$

resulting after a change to spherical variables in

6 $$\begin{aligned} \int _{\partial \varOmega } Y^{l,m} u Z \, \mathrm {d}\varOmega , \end{aligned}$$

where the generalized density of states *Z* is obtained from the Jacobian of the coordinate transformation as

7 $$\begin{aligned} Z(\varepsilon , \theta , \varphi ) = \frac{k^2}{(2\pi )^3} \frac{\partial k}{\partial \varepsilon } . \end{aligned}$$

This generalized density of states differs from the conventional density of states by a factor of $4\pi$, which is obtained in the spherically symmetric case by an integration over the angles $\theta$ and $\varphi$. The important detail in (6) is the generalized density of states entering the integrand. If the generalized density of states is modeled as spherically symmetric, i.e., $Z=Z(\varepsilon )$, then (4) and (6) differ only by a constant factor for fixed kinetic energy $\varepsilon$. On the other hand, a full angular dependence of *Z* will lead to unrelated expansion coefficients obtained from (4) and (6) in general.

Since the distribution function *f* is a-priori unknown and only known to fulfill the BTE, it is not enough to only compute projections of the form (4) or (6). Instead, a system of equations for the unknown expansion coefficients $f_{l,m}$ needs to be derived from the BTE. Such a system is obtained by projecting (2) onto the spherical harmonics $Y^{l,m}$. For details of the derivation, we refer to the literature [40] and directly state the resulting set of equations:

8 $$\begin{aligned}&\frac{\partial g_{l,m}}{\partial t} + \frac{\partial (\varvec{F} \cdot \hat{\varvec{j}}_{l,m})}{\partial \varepsilon } + \nabla _{\varvec{x}} \cdot \hat{\varvec{j}}_{l,m} \nonumber \\&\quad -\, \hat{T} \varvec{F} \cdot \varvec{\varGamma }_{l,m} = Q_{l,m}\{ g \} , \end{aligned}$$

where we set $g := fZ$ motivated by (6), $\hat{\varvec{j}}$ is the generalized current density given by

9 $$\begin{aligned} \hat{\varvec{j}}_{l,m} = \int _{\partial \varOmega } \hat{T} \varvec{v} g Y^{l,m} \, \mathrm {d}\varOmega , \end{aligned}$$

and

10 $$\begin{aligned} \varvec{\varGamma }_{l,m} = \int _{\partial \varOmega } \frac{g}{\hbar k} \left( \frac{\partial Y^{l,m}}{\partial \theta } \varvec{e}_{\theta } + \frac{1}{\sin \theta } \frac{\partial Y^{l,m}}{\partial \varphi } \varvec{e}_{\varphi } \right) \, \mathrm {d}\varOmega \end{aligned}$$

with unit vectors $\varvec{e}_{\theta }$ and $\varvec{e}_{\varphi }$ in the spherical coordinate system for the $\theta$ and $\varphi$ directions, respectively. The projected scattering operator $Q_{l,m}\{ g \}$ will be discussed below. To better expose the structure of the equations, we combine $\nabla _{\varvec{x}}$ and $\partial /\partial \varepsilon$ to yield a divergence in $(\varvec{x}, \varepsilon )$-space:

11 $$\begin{aligned} \frac{\partial g_{l,m}}{\partial t} + \nabla _{\varvec{x}, \varepsilon } \cdot \tilde{\varvec{j}}_{l,m} - \hat{T} \varvec{F} \cdot \varvec{\varGamma }_{l,m} = Q_{l,m}\{ g \} , \end{aligned}$$

with

12 $$\begin{aligned} \tilde{\varvec{j}}_{l,m} = \left( \begin{array}{c} \hat{\varvec{j}}_{l,m} \\ \varvec{F} \cdot \hat{\varvec{j}}_{l,m} \end{array} \right) . \end{aligned}$$

**Fig. 1:**
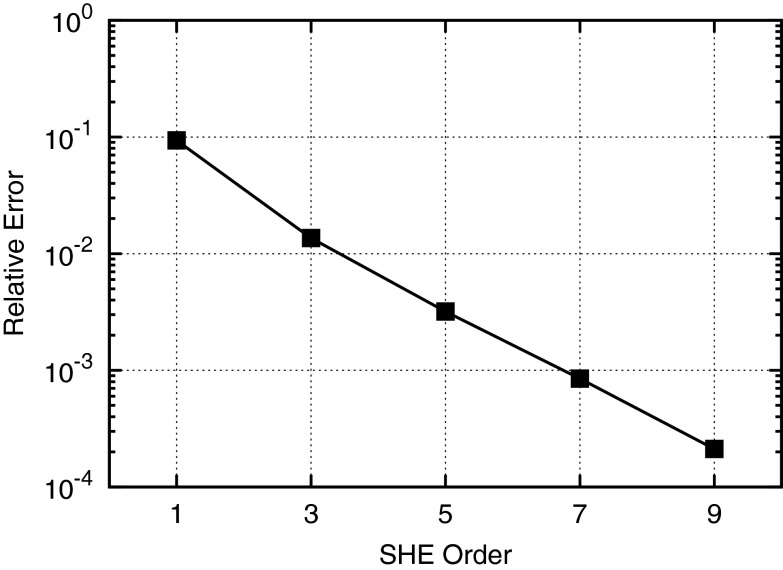
Comparison of the relative error in the collector current of a silicon-germanium heterojunction bipolar transistor (base thickness: 24 nm) for different SHE orders [44]. First-order expansions show an error of 10 % compared to an eleventh-order expansion, hence expansion orders of three to seven are more appropriate for scaled-down devices

Similar to numerical solution techniques based on Fourier series, we substitute a SHE truncated at finite expansion order $l_{\max }$ for *g* as

13 $$\begin{aligned} g \approx \sum _{l^\prime =0}^{l^\prime _{\max }} \sum _{m^\prime =-l^\prime }^{l^\prime } g_{l^\prime ,m^\prime } Y^{l^\prime ,m^\prime } . \end{aligned}$$

Typical values for $l_{\max }$ for scaled-down devices are between three to seven, resulting in practically negligible relative truncation errors for relevant macroscopic quantities. For example, the relative error plotted in Fig. 1 for the collector current of a silicon-germanium heterojunction bipolar transistor is significant (10 %) when comparing a first-order expansion to an eleventh-order expansion. In contrast, a third-order expansion with an error of only one percent is sufficient for most practical purposes.

With a truncated SHE and Einstein’s summation convention for repeated upper and lower indices, we obtain

14 $$\begin{aligned}&\hat{\varvec{j}}_{l,m} = \hat{\varvec{v}}_{l,m}^{l^\prime , m^\prime } g_{l^\prime , m^\prime } , \end{aligned}$$

15 $$\begin{aligned}&\varvec{\varGamma }_{l,m} = \varvec{\varGamma }_{l,m}^{l^\prime , m^\prime } g_{l^\prime , m^\prime } \end{aligned}$$

with

16 $$\begin{aligned}&\hat{\varvec{v}}_{l,m}^{l^\prime , m^\prime }= \int _{\partial \varOmega } \hat{T} \varvec{v} Y^{l^\prime , m^\prime } Y^{l,m} \, \mathrm {d}\varOmega ,\end{aligned}$$

17 $$\begin{aligned}&\varvec{\varGamma }_{l,m}^{l^\prime , m^\prime }= \int _{\partial \varOmega } \frac{Y^{l^\prime , m^\prime }}{\hbar k} \left( \frac{\partial Y^{l,m}}{\partial \theta } \varvec{e}_{\theta } + \frac{1}{\sin \theta } \frac{\partial Y^{l,m}}{\partial \varphi } \varvec{e}_{\varphi } \right) \, \mathrm {d}\varOmega . \end{aligned}$$

Before applying the truncated expansion to the scattering operator, we split the scattering operator into contributions from in-scattering (a carrier enters the considered trajectory) and out-scattering (a carrier leaves the considered trajectory) via

$$\begin{aligned} Q\{g\} = \sum _\eta Q^{\mathrm {in}}_{\eta }\{g\} - Q^{\mathrm {out}}_{\eta } \{g\} , \end{aligned}$$

where the individual scattering processes are identified through the index $\eta$. This allows us to separate evaluations of the distribution function at other energies (in-scattering) than the energy of the respective trajectory (out-scattering).

Substituting the truncated expansion (13) into the system of projected BTEs (8) result in

18 $$\begin{aligned}&\frac{\partial g_{l,m}}{\partial t} + \nabla _{\varvec{x},\varepsilon } \cdot \tilde{\varvec{j}}_{l,m}^{l^\prime , m^\prime } g_{l^\prime ,m^\prime } - \varvec{F} \cdot \varvec{\varGamma }_{l,m}^{l^\prime , m^\prime } g_{l^\prime ,m^\prime }\nonumber \\&\quad =\sum _{\eta } \left[ Q_{\eta ; l,m}^{\mathrm {in}; l^\prime , m^\prime }g_{l^\prime , m^\prime }(\varvec{x}, \varepsilon \mp \hbar \omega _{\eta }, t)- Q_{\eta ; l,m}^{\mathrm {out}; l^\prime ,m^\prime }g_{l^\prime , m^\prime } \right] , \end{aligned}$$

where the inelastic energy transfer involved in the scattering process identified by $\eta$ is $\hbar \omega _{\eta }$. Equation 18 defines a system of $(l_{\max } + 1)^2$ coupled linear partial differential equations of first order, where shifted arguments $\varepsilon \mp \hbar \omega _{\eta }$ appear in the in-scattering terms. The system is posed in the five-dimensional $(\varvec{x}, \varepsilon , t)$-space rather than the original seven-dimensional $(\varvec{x}, \varvec{k}, t)$-space of the BTE. For stationary simulations, the solution space further reduces to four dimensions (or three and two dimensions for two- or one-dimensional device simulations, respectively). Therefore, solutions for the unknown expansion coefficients $g_{l,m}$ are much cheaper to compute in general than full solutions *f* of the BTE.

### Boundary conditions

The system of equations (18) needs to be supplemented with suitable boundary conditions in order to fully specify the system. Homogeneous Neumann boundary conditions are imposed at spatial non-contact boundaries. Similarly, homogeneous Neumann boundary conditions are applied at the lower energy boundary at $\varepsilon = 0$ and for the upper energy boundary at $\varepsilon = \varepsilon _{\max }$ for some user-defined value of $\varepsilon _{\max }$. Scattering processes with initial or final energy outside the considered energy range, including scatter events to or from the band gap, are invalid and hence ignored.

Early publications imposed Maxwell-Boltzmann distributions $f^{\mathrm {eq}}$ via Dirichlet boundary conditions of the form

19 $$\begin{aligned} f_{l,m}(\varepsilon ) = \left\{ \begin{array}{l@{\quad }l} f^{\text {eq}} := M \exp \bigl ( \frac{\varepsilon }{k_{\mathrm {B}}T} \bigr ) , &{} l=m=0 , \\ 0 , &{} \mathrm {otherwise} , \end{array} \right. \end{aligned}$$

at the contacts, where $k_{\mathrm {B}}$ is the Boltzmann constant, *T* denotes temperature, and *M* is a suitable normalization factor in order to obtain the correct contact carrier density. This is in some sense similar to contact models often used for moment-based models, where a known value of the carrier density is prescribed as a Dirichlet boundary condition. At closer inspection, however, Maxwell-Boltzmann distributions as Dirichlet boundary conditions for SHE are problematic: While such a thermal equilibrium assumption is reasonable at the inflow contacts, it leads to steep gradients (so-called boundary layers) at the outflow-contact at higher bias [22]. In other words, a heated carrier distribution is forced to thermal equilibrium at the outflow-contact.

Generation/recombination processes solve the issues with boundary layers at Dirichlet boundaries. For example, Jungemann et al. imposed the volume rate

20 $$\begin{aligned} \gamma _{l,m} = - \frac{g_{l,m} - Z_{l,m}f^{\text {eq}}}{\tau _0} , \end{aligned}$$

as volume sources at the contacts, where $Z_{l,m}$ is the spherical harmonics expansion coefficient of the generalized density of states, $f_{\text {eq}}$ is the equilibrium (Maxwell-Boltzmann) distribution as in (19), and $\tau _0$ is the recombination time [22, 40]. Here, $\tau _0$ provides control over the difference between thermal equilibrium and the computed solution. In the limit $\tau _0 \rightarrow 0$, the Dirichlet boundary condition (19) is recovered.

Hong et al. proposed a parameter-free improvement of (20) [41]. The new model is based on a surface generation rate of the form

21 $$\begin{aligned} \gamma ^{\mathrm {s}}(\varvec{k}^\prime )= & {} - \bigl [f^{\text {eq}} \mathbbm {1}_{[0, \infty )}(-\hat{T}\varvec{v} \cdot \varvec{n})\nonumber \\&+ \,f \mathbbm {1}_{[0, \infty )}(\hat{T}\varvec{v} \cdot \varvec{n}) \bigr ] \hat{T} \varvec{v} \cdot \varvec{n} \end{aligned}$$

with outward-pointing unit normal vector $\varvec{n}$ at the contact and the Heaviside step function $\mathbbm {1}_{[0, \infty )}$. Here, the first term describes carriers in thermal equilibrium entering the device ($\hat{T}\varvec{v}$ needs to point into the device), while the second term describes the annihilation of heated carriers leaving the device. Such a boundary condition corresponds to a thermal bath contact as used in Monte Carlo simulations [3].

### Stabilization and H-transform

The partial derivatives with respect to the spatial variable $\varvec{x}$ and the kinetic energy $\varepsilon$ describe the motion of carriers in free flight. In the absence of scattering mechanisms, carriers solely gain or lose kinetic energy in reaction to the force term $\varvec{F}$. Therefore, the trajectories of carriers in free flight in $(\varvec{x}, \varepsilon )$-space mirror the potential profile throughout the device. Regular discretizations with respect to the kinetic energy $\varepsilon$ are unable to trace these trajectories accurately, because particles in free flight gain and lose kinetic energy in response to the externally applied field. As a consequence, numerical instabilities develop in regions of high electric field, unless numerical stabilization leading to a stable discretization as discussed in Sect. 4 is applied. Rahmat et al. used a semi-empirical upwind scheme to stabilize the SHE equations for device simulation in the micrometer regime [27]. Jungemann et al. applied the maximum entropy dissipation scheme (MEDS) [37] and obtained good numerical stability for devices of about 100 nm length [40]. They multiplied the equations obtained for odd *l* with

22 $$\begin{aligned} \exp \left( \frac{\varepsilon + \varepsilon _{\mathrm {b}} + \mathrm {q}\psi (\varvec{x})}{k_{\mathrm {B}} T} \right) \end{aligned}$$

in order to address the exponential decay of the distribution function with kinetic energy and to better follow the trajectory of carriers in free flight. This ultimately results in a Scharfetter-Gummel-like stabilization of the discrete system.

Ballistic transport becomes increasingly dominant for smaller devices, so in addition to MEDS the so-called *H*-transformation [16] was applied in [41] and used in all subsequent publications. The essence of the *H*-transformation is to apply a change of coordinates from kinetic energy $\varepsilon$ to total energy $H = \varepsilon + \varepsilon _{\mathrm {b}} + \mathrm {q}\psi (\varvec{x})$, through which the derivative with respect to energy in (18) vanishes. Overall, one obtains

23 $$\begin{aligned}&\frac{\partial g_{l,m}}{\partial t} + \mathrm {q} \frac{\partial g_{l,m}}{\partial H}\frac{\partial \psi }{\partial t} + \nabla _{\varvec{x}} \cdot \hat{\varvec{j}}_{l,m}^{l^\prime , m^\prime } g_{l^\prime ,m^\prime } - \hat{T} \varvec{F} \cdot \varvec{\varGamma }_{l,m}^{l^\prime , m^\prime } g_{l^\prime ,m^\prime }\nonumber \\&\quad = \sum _{\eta } \left[ Q_{\eta ; l,m}^{\mathrm {in}; l^\prime , m^\prime }g_{l^\prime , m^\prime }(\varvec{x}, H \mp \hbar \omega _{\eta }, t) - Q_{\eta ; l,m}^{\mathrm {out}; l^\prime ,m^\prime }g_{l^\prime , m^\prime } \right] . \end{aligned}$$

For simplicity, all variable names were reused in (23), even though all quantities are now a function of $(\varvec{x}, H, t)$ instead of $(\varvec{x}, \varepsilon , t)$. It is important to note that the variable transformation results in an additional term containing the derivative of the SHE expansion coefficients with respect to total energy *H* and the time derivative of the potential. While the term does not contribute for stationary solutions, it is important to consider the term for small-signal analysis as well as noise simulations [45].

**Fig. 2:**
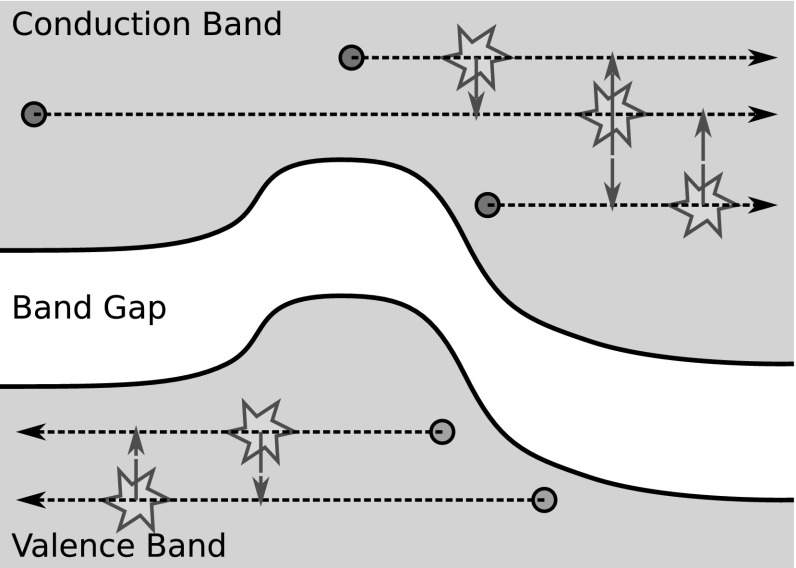
Carrier trajectories (*dotted horizontal lines*) in free flight are given by constant total energy *H*. Scattering mechanisms couple the individual trajectories (*vertical arrows*). The shape of the band edge is determined by the material configuration and the electrostatic potential

After applying the *H*-transformation, the carrier trajectories in free flight, which are given by constant total energy *H*, are well resolved when using a regular grid with respect to the total energy coordinate, cf. Fig. 2. The price to pay for the improved numerical stability is the dependence of the band edge on the potential; simulation regions for the conduction and valence band edges must be recomputed after each change of the potential, resulting in stability issues for transient simulations, cf. Sect. 6.3. MEDS applied to the *H*-transformed equations results in the multiplication of equations for odd *l* by a constant, hence this constant can also be omitted without changing the solution of the system. As discussed by Hong et al. favorable numerical properties are obtained when using the adjoint equations for the discretization instead [41, 46]. The adjoint equations can also be obtained through direct manipulation and read

24 $$\begin{aligned}&\frac{\partial g_{l,m}}{\partial t} + \mathrm {q} \frac{\partial g_{l,m}}{\partial H}\frac{\partial \psi }{\partial t} + \hat{\varvec{j}}_{l,m}^{l^\prime , m^\prime } \cdot \nabla _{\varvec{x}} g_{l^\prime ,m^\prime } + \hat{T} \varvec{F} \cdot \varvec{\varGamma }_{l^\prime , m^\prime }^{l,m} g_{l^\prime ,m^\prime }\nonumber \\&\quad = \sum _{\eta } \left[ Q_{\eta ; l,m}^{\mathrm {in}; l^\prime , m^\prime }g_{l^\prime , m^\prime }(\varvec{x}, H \mp \hbar \omega _{\eta }, t)- Q_{\eta ; l,m}^{\mathrm {out}; l^\prime , m^\prime }g_{l^\prime , m^\prime } \right] , \end{aligned}$$

where compared to (23) the divergence of the generalized current density was transformed to a gradient of the generalized distribution function coefficients $g_{l^\prime , m^\prime }$, the sign of the term involving the force $\varvec{F}$ is flipped, and the indices of $\varGamma _{l,m}^{l^\prime , m^\prime }$ are changed to $\varGamma _{l^\prime , m^\prime }^{l,m}$.

**Fig. 3:**
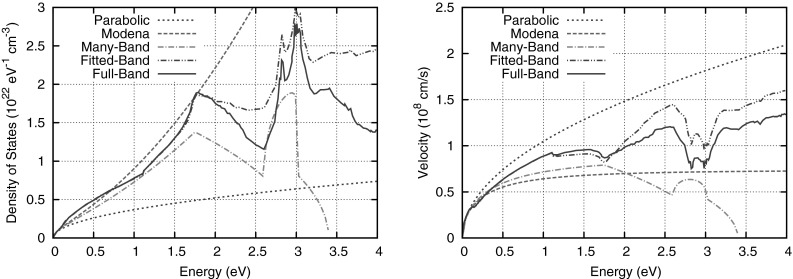
Comparison of the density of states *Z* and the group velocity *v* for different dispersion relations commonly used with the SHE method [47–51]. The original parameter set for the many-band model from [48] is based on different full-band data than the full-band data plotted here, which explains the relatively large deviations

## Modeling

In this section, we discuss material-specific properties and physical details to extend the general description of the SHE method in Sect. 2. These details are essential for predictive device simulation and require a careful modeling of the underlying material. While most of the discussion is centered around silicon and germanium, the concepts are likely to be applicable to other materials as well, even though other processes such as polar-optical phonon scattering may play a much more important role.

### Band structure

From the dispersion relation $\varepsilon (\varvec{k})$, one can fully describe the ballistic transport of carriers in a device. More precisely, both the group velocity $\varvec{v} = \nabla _{\varvec{x}}\varepsilon / \hbar$ and the density of states (7) are directly obtained. During the derivation of the SHE method it is required that the mapping from $\varepsilon$ to *k* is one-to-one, hence the term $(\hbar k)^{-1}$ in (10) can be evaluated directly. For common analytical bandstructure models, namely the parabolic bandstructure

25 $$\begin{aligned} \varepsilon (\varvec{k}) = \frac{\hbar ^2 k^2}{2m^*} \qquad \mathrm {(parabolic)} \end{aligned}$$

with effective mass $m^*$ and the non-parabolic modification [47, 49]

26 $$\begin{aligned} \varepsilon (1 + \alpha \varepsilon ) = \frac{\hbar ^2 k^2}{2m^*} \qquad \mathrm {(nonparabolic)} \end{aligned}$$

also known as Modena model or Kane model, these one-to-one mappings are obtained directly. Similarly, Brunetti et al. proposed to invert the dispersion relation for each band of the many-band model for silicon [48]. These models typically reproduce the density of states as well as the group velocity fairly well at energies below 1 eV, but fail to provide good approximations at higher energies.

A better approximation of the dispersion relation can in principle be obtained from a SHE of the inverse dispersion relation

27 $$\begin{aligned} k(\varepsilon , \theta , \varphi ) = \sum _{l=0}^{l^{\mathrm k}_{\max }} \sum _{m=-l}^l k_{l,m} Y^{l,m} \end{aligned}$$

for some maximum expansion order $l^{\mathrm k}_{\max }$. Such an approach was pursued by Kosina et al. for the valence band [43] up to an energy of 1.27 eV and later refined by Pham et al. [52]. A fitted band structure based on the SHE for the conduction band was developed by Matz et al. [50, 51]. However, a systematic error cannot be avoided because of the requirement of a one-to-one mapping between kinetic energy $\varepsilon$ and the modulus of the wave vector *k*.

Additional full-band effects can be considered by relaxing the requirement of a one-to-one mapping for the modulus of the wave vector *k* and the density of states *Z*. Vecchi et al. found that for a first-order SHE the equations can be recast such that the term $\varvec{\varGamma }_{l,m}$ as defined in (10) does not contribute and hence an explicit form is not required for *k* [23]. Thus, even though such a one-to-one mapping is formally required for the derivation of the SHE method, full-band data without an explicit one-to-one mapping such as in Fig. 3 can be used directly. Jin et al. extended this approach to expansions of arbitrary order [53]. They observed that under the assumption of spherically symmetric dispersion relations one can write

28 $$\begin{aligned} 2\frac{Z}{\hbar k} = \frac{\partial vZ}{\partial \varepsilon } \end{aligned}$$

and eliminate the explicit dependence on *k* in (10). With this, Jin et al. showed that full-band data can directly be used for the group velocity *v* and the generalized density of states *Z*. They demonstrated good agreement of the distribution function obtained from one-dimensional device simulations using the SHE method with results from full-band Monte Carlo simulations.

Hong et al. proposed a further refinement of the approach by Jin et al. by postponing the isotropic valley approximations in earlier approaches until the last stage of the model derivation [46]. The proposed method is to use a generalized coordinate transformation to construct a model of the first conduction band for increased accuracy, while higher conduction bands are approximated using the isotropic model. This hybrid approach constitutes a good compromise between higher accuracy and lower computational cost.

### Pauli principle

The scattering operator for events other than carrier-carrier scattering is often written in the low-density approximation as

29 $$\begin{aligned} Q\{f\}= & {} \frac{1}{(2\pi )^3} \int _{\mathcal {B}} s(\varvec{x}, \varvec{k}^*, \varvec{k})f(\varvec{x}, \varvec{k}^*, t)\nonumber \\&-\, s(\varvec{x}, \varvec{k}, \varvec{k}^*)f(\varvec{x}, \varvec{k}, t)\, \mathrm {d}\varvec{k}^* \end{aligned}$$

with scattering rate $s(\varvec{x}, \varvec{k}^{\mathrm {initial}}, \varvec{k}^{\mathrm {final}})$ for a scattering process from an initial state $\varvec{k}^{\mathrm {initial}}$ to a final state $\varvec{k}^{\mathrm {final}}$. The first term in the integrand refers to in-scattering, while the second term in the integrand denotes out-scattering. The low-density approximation is justified whenever the term $(1 - f)$ at the final state, i.e., the probability of the final state being vacant, can be approximated by 1. However, this approximation can no longer justified at medium to high carrier densities and the Pauli exclusion principle needs to be considered via the full scattering operator:

30 $$\begin{aligned} Q\{f\}= & {} \frac{1}{(2\pi )^3} \int _{\mathcal {B}} s(\varvec{x}, \varvec{k}^*, \varvec{k})f(\varvec{x}, \varvec{k}^*, t)\bigl (1 - f(\varvec{x}, \varvec{k}, t)\bigr )\nonumber \\&-\, s(\varvec{x}, \varvec{k}, \varvec{k}^*)f(\varvec{x}, \varvec{k},t)\bigl (1 - f(\varvec{x}, \varvec{k}^*, t)\bigr ) \, \mathrm {d}\varvec{k}^* \end{aligned}$$

**Fig. 4:**
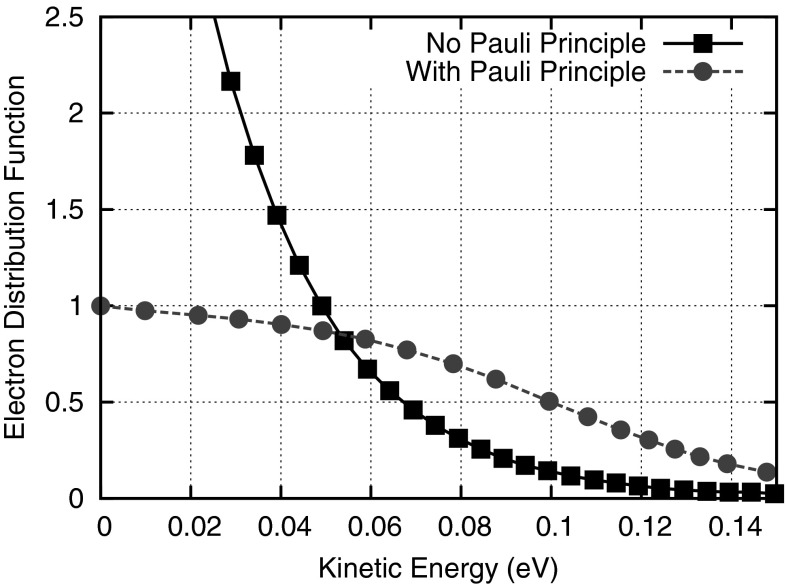
Comparison of the electron distribution function for a silicon-germanium heterojunction bipolar transistor at room temperature with a maximum doping level of $2 \times 10^{20}\,\mathrm {cm}^{-3}$ in the emitter region [54]. If the Pauli principle is not considered, the distribution function obtains values higher than unity at such extreme dopings

Thus, the linear system of SHE equations in the low-density approximation becomes nonlinear if the Pauli exclusion principle is included. This weak nonlinearity, however, is only a mild concern for simulations with frozen field; self-consistent simulations need to account for the nonlinear coupling of the SHE equations to the Poisson equation. Hong et al. investigated the influence of Pauli’s exclusion principle and found a notable difference only for doping concentrations above $10^{18}$ cm$^{-3}$ [54]. Figure 4 depicts the influence of Pauli’s exclusion principle on the electron distribution function in a highly doped silicon-germanium heterojunction bipolar transistor. Considerable influence of Pauli’s exclusion principle is only observed at energies below 0.2 eV. Without Pauli’s exclusion principle, values higher than unity are obtained, which do not make sense from a mathematical and physical point of view.

**Fig. 5:**
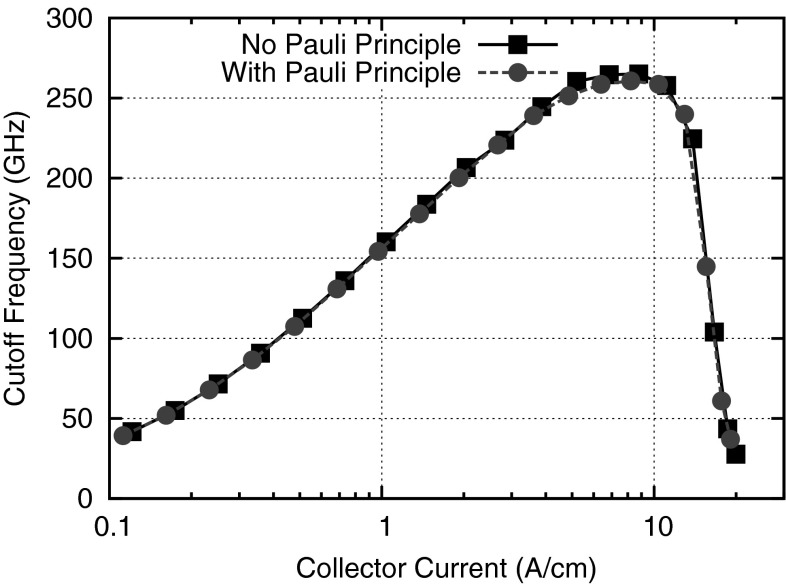
Comparison of the cutoff frequency in a silicon-germanium heterojunction bipolar transistor computed with and without Pauli principle for a collector–emitter bias of 1.2 Volt [54]. The difference is at most two percent, which is negligible for most practical applications

A practical evaluation of the influence of Pauli’s exclusion principle is given in Fig. 5, where the simulated cutoff frequencies for a silicon-germanium heterojunction bipolar transistor are compared. Even though there is a high doping level of $2 \times 10^{20} \mathrm {cm}^{-3}$ in the emitter region of the device, the difference in the computed cutoff frequency of only two percent shows that the influence of Pauli’s exclusion principle is small. The difference is small because macroscopic quantities such as the electron density remain essentially unchanged whether or not Pauli’s exclusion principle is considered. In this case, the cutoff frequency is mainly determined by the electron distribution function just above the energy barrier in the base region, where the impact of the Pauli principle is negligible. Consequently, it is often acceptable to ignore Pauli’s exclusion principle for simulating device characteristics.

**Fig. 6:**
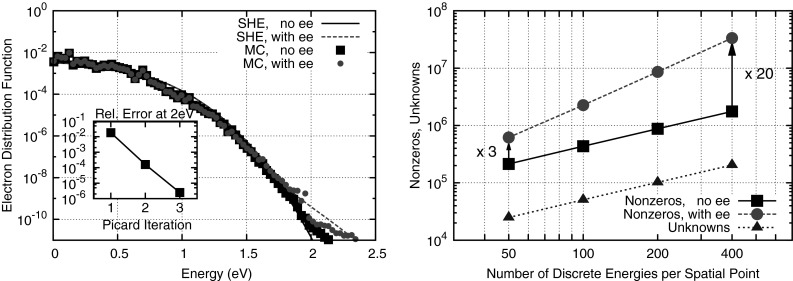
*Left* Comparison of the electron distribution with and without electron-electron scattering in a bulk semiconductor at 100 kV/cm including a convergence plot of the electron distribution function at 2 eV when including electron-electron scattering. *Right* Exemplary number of nonzeros in the system matrix with and without electron-electron scattering in comparison to the number of unknowns for a MOSFET simulation. [55]

### Carrier-carrier scattering

In addition to Pauli’s exclusion principle, the scattering operator in (29) also becomes nonlinear if carrier-carrier interaction is considered. Using a low-density approximation, the scattering operator becomes

31 $$\begin{aligned} Q\{f\}= & {} \frac{1}{(2\pi )^3} \int _{\mathcal {B}} s(\varvec{x}, \varvec{k}^*, \varvec{k}, \varvec{k}_2^*, \varvec{k}_2) f(\varvec{x}, \varvec{k}^*, t) f(\varvec{x}, \varvec{k}_2^*, t)\nonumber \\&-\, s(\varvec{x}, \varvec{k}, \varvec{k}^*, \varvec{k}_2, \varvec{k}_2^*) f(\varvec{x}, \varvec{k}, t) f(\varvec{x}, \varvec{k}_2, t) \, \mathrm {d}(\varvec{k}^*, \varvec{k}_2, \varvec{k}_2^*) . \end{aligned}$$

This quadratic nonlinearity in the distribution function of the scattering operator becomes a forth-order nonlinearity if Pauli’s exclusion principle is considered as well. The scattering rate $s(\varvec{x}, \varvec{k}, \varvec{k}^*, \varvec{k}_2, \varvec{k}_2^*)$ for simultaneous transitions of two carriers from $\varvec{k}$ to $\varvec{k}^*$ and $\varvec{k}_2$ to $\varvec{k}_2^*$ needs to account for both energy and momentum conservation, i.e.,

32 $$\begin{aligned}&s(\varvec{x}, \varvec{k}, \varvec{k}^*, \varvec{k}_2, \varvec{k}_2^*) \nonumber \\&\quad \sim \delta (\varvec{k} + \varvec{k}_2 - \varvec{k}^* - \varvec{k}_2^*) \delta (\varepsilon + \varepsilon _2 - \varepsilon ^* - \varepsilon _2^*) \end{aligned}$$

with Dirac distribution $\delta$. This dual conservation property induces additional complications when compared to e.g., phonon scattering processes, where only energy needs to be conserved. Moreover, the strong angular anisotropy of the scattering rate needs to be resolved appropriately. Ventura et al. developed a technique for simulating carrier-carrier scattering using a first-order SHE method [30, 56]. Rupp et al. refined the method to arbitrary SHE order and verified the approach for bulk silicon through comparison with Monte Carlo results [55]. A rigorous calibration for device simulation is, however, extremely difficult, since measurement data for the carrier distribution function are not available for comparison.

The inclusion of carrier-carrier scattering increases the computational effort considerably. This is due to the non-local coupling of the carrier-carrier scattering operator with respect to energy. In other words, for a fixed spatial coordinate $\varvec{x}$, carrier-carrier scattering may occur between two carriers with arbitrary initial and final energies. In contrast, carrier-phonon scattering involves a fixed energy transfer only. As a consequence, execution times as well as memory requirements when considering carrier-carrier scattering increase by about one to two orders of magnitude depending on the resolution with respect to energy, cf. Fig. 6.

### Generation and recombination

First publications on the SHE method have considered one carrier type only. Later, the BTE was coupled with a continuity equation for the second carrier type (e.g., [19]). Rupp et al. applied the SHE method to both carrier types and included transitions between the valence and the conduction band. They modeled the coupling of the two BTEs by generation and recombination processes via trap levels in the band gap [57], exactly reproducing the Shockley-Read-Hall model in the macroscopic limit. Other processes such as impact ionization may also contribute to carrier generation or recombination [58]. Because of the importance of Shockley-Read-Hall-like generation and recombination process in silicon, we will consider generation and recombiation via traps in more detail in the following.

**Fig. 7:**
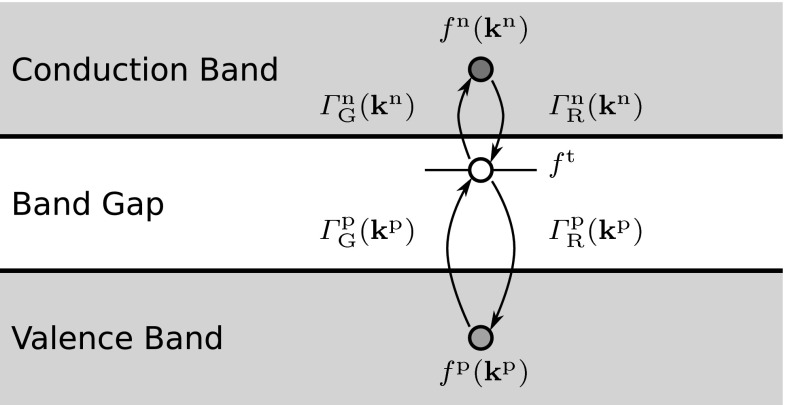
Generation or recombination of an electron–hole pair through a trap in the band gap

**Fig. 8:**
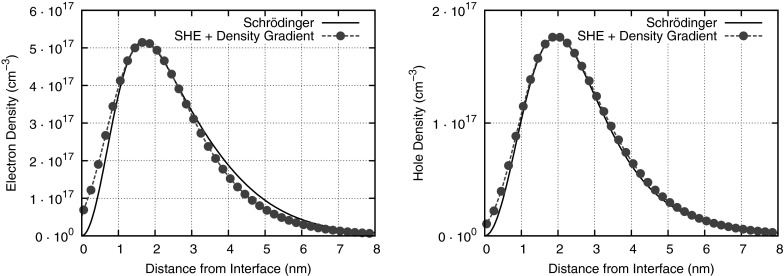
Comparison of carrier densities obtained for the simulation of an nMOS structure (*left*) and a pMOS structure (*right*). Each MOS structure has a length of 100 nm and acceptor and donor doping densities of $3 \times 10^{16}$ cm$^{-3}$, respectively

More formally, a BTE is considered for the distribution functions $f^{\mathrm {n}}$ and $f^{\mathrm {p}}$ for electrons and holes, respectively. The additional term

33 $$\begin{aligned} (1 - f^{\mathrm {n}}) \varGamma ^{\mathrm {n}}_{\mathrm {G}} N^{\mathrm {t}} f^{\mathrm {t}} - f^{\mathrm {n}} \varGamma ^{\mathrm {n}}_{\mathrm {R}} N^{\mathrm {t}} (1 - f^{\mathrm {t}}) \end{aligned}$$

in the BTE models the generation (with rate $\varGamma ^{\mathrm {n}}_{\mathrm {G}}$) and recombination (with rate $\varGamma ^{\mathrm {n}}_{\mathrm {R}}$) of an electron in the conduction band via a trap $\mathrm {t}$ with trap density $N^{\mathrm {t}}$ and occupation probability $f^{\mathrm {t}}$, cf. Fig. 7. Similarly, the generation and recombination of a hole in the valence band is modeled through the additional term

34 $$\begin{aligned} (1 - f^{\mathrm {p}}) \varGamma ^{\mathrm {p}}_{\mathrm {G}} N^{\mathrm {t}} (1 - f^{\mathrm {t}}) - f^{\mathrm {p}} \varGamma ^{\mathrm {p}}_{\mathrm {R}} N^{\mathrm {t}} f^{\mathrm {t}} . \end{aligned}$$

The trap occupation probability $f^{\mathrm {t}}$ can be computed explicitly from the electron and hole distribution functions [57].

### Quantum mechanical corrections

If the SHE method is used for the simulation of scaled-down devices in the deca-nanometer regime, the semi-classical nature of the BTE is not enough to account for quantum mechanical effects. In particular, quantum mechanics requires that the peak carrier concentration in the channel of a MOSFET is located a few nanometers away from the interface to the gate oxide rather than at the interface. A solution of the Boltzmann-Poisson system, however, does not reflect this fact unless special correction schemes are employed.

The density gradient model is a popular method to capture quantum mechanical effects to first order by extending the drift-diffusion model [59, 60]. Related approaches for including quantum mechanical corrections in other moment-based methods also exist, see for example [61–63]. Bina extended the first-order SHE method such that quantum mechanical corrections provided by the density gradient model are considered [64]. Similar to how the density gradient model is obtained from the drift-diffusion model, he introduced a correction potential $\gamma (\varvec{x}, t)$, which fulfills

$$\begin{aligned} \gamma (\varvec{x}, t) = \frac{\hbar ^2}{12\lambda k_{\mathrm {B}} T_{\mathrm {L}} m^*} \bigl (\varDelta \psi + \varDelta \gamma \bigr ) \end{aligned}$$

with fitting parameter $\lambda$, Boltzmann constant $k_{\mathrm {B}}$, lattice temperature $T_{\mathrm {L}}$, effective mass $m^*$, and electrostatic potential $\psi$. Homogeneous Dirichlet boundary conditions are employed at the contacts, whereas Robin boundary conditions of the form

$$\begin{aligned} \alpha \gamma + \beta \frac{\partial \gamma }{\partial \varvec{n}} = f \end{aligned}$$

with constants $\alpha$, $\beta$, *f*, and outer normal vector $\varvec{n}$ are used at semiconductor-insulator interfaces.

Simulation results show good quantitative agreement with solutions of the Schrödinger equation for one-dimensional simulations of metal-oxide-semiconductor structures, cf. Fig. 8. In practice, these corrections come at negligible cost, because the additional numerical effort for computing the quantum mechanical correction potential is tiny compared to the numerical effort required for computing the SHE coefficients.

Subband-splitting is another way of considering quantum mechanical effects. A two- or one-dimensional BTE is solved in transport direction, while the one- or two-dimensional Schrödinger equation is solved in the perpendicular confinement directions, respectively. Instead of the SHE method, Fourier expansions for solving the BTE with a two-dimensional momentum space are sufficient, hence reducing the computational effort [65, 66]. A degenerate BTE with one-dimensional momentum space can even be solved directly without any problems. As a consequence, we will not discuss these approaches in more detail, but refer to the literature for further details [46, 67].

## Numerics

The presentation of the SHE method in Sect. 2 as well as the various models discussed in Sect. 3 was based on a continuous formulation of the equations. In this section, we outline the discretization of the *H*-transformed SHE equations (23), discuss solution procedures for self-consistency with Poisson’s equation, and discuss numerical tweaks to minimize execution times.

### Discretization

The finite volume method (also known as box integration method) is an appealing choice for the discretization of the *H*-transformed SHE equations in (23), because it ensures local charge conservation properties similar to moment-based models. In a naive discretization, all expansion coefficients $g_{l,m}$ are discretized in a conforming manner and the spatial divergence is converted to a surface integral as usual. Such a direct discretization, however, suffers from spurious numerical oscillations and instabilities.

To understand the numerical instability of conforming discretizations, consider the coupling nature of the terms $\varvec{j}_{l,m}^{l^\prime , m^\prime }$ and $\varvec{\varGamma }_{l,m}^{l^\prime , m^\prime }$. Because of symmetries of the underlying physical processes, these coupling terms vanish whenever *l* and $l^\prime$ are of the same parity [37, 40]. Moreover, due to the derivatives with respect to the angles in (17), there holds

35 $$\begin{aligned} \varvec{\varGamma }_{0,0}^{l^\prime , m^\prime } = \varvec{0} , \end{aligned}$$

hence the ballistic flight of carriers is entirely described by the term $\nabla _{\varvec{x}} \cdot \hat{\varvec{j}}_{l,m}$ for a first-order SHE method. Note that this is in analogy to the drift-diffusion equations, where the divergence is responsible for the current conservation. Due to the even-to-odd and odd-to-even coupling structure of $\varvec{j}_{l,m}^{l^\prime , m^\prime }$, the expansion coefficients $g_{1,m}$ can be readily identified with the currents. Even-order expansion coefficients describe densities, therefore it is advantageous to arrange the discrete unknowns such that adjacent even-order expansion coefficients are coupled via odd-order expansion coefficients describing the flux between the two. In a finite volume method, even-order unknowns (i.e.,  $g_{l,m}$ with *l* even) are thus associated with the discrete control volumes, while odd-order unknowns are associated with the interfaces between control volumes. For a discretization based on kinetic energy, the odd-order unknowns are associated with the corners of the control volume interfaces in order to account for the additional energy derivative [40]. A discretization of the *H*-transformed equations, however, directly associates the odd-order unknowns with each interface at the same total energy *H*. The reason is that derivatives with respect to energy are absent and thus a staggered grid with respect to energy is not appropriate [68].

**Fig. 9:**
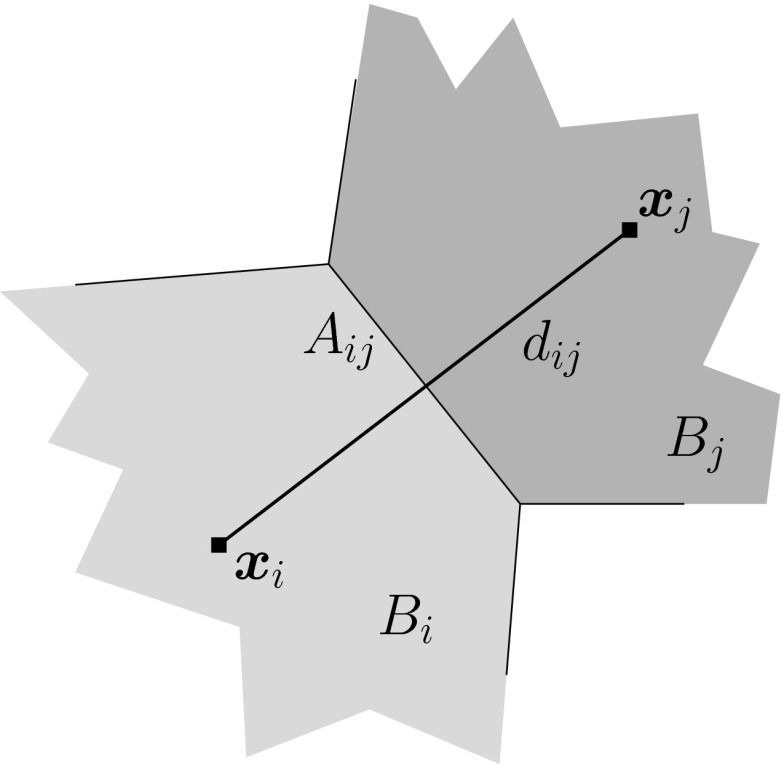
Schematic of the notation used for the finite volume discretization

The most commonly used finite volume scheme for semiconductor device simulation is vertex-based. Control volumes (“boxes”) are taken from the dual Voronoi grid of a Delaunay mesh so that each box can be associated with a vertex and vice versa. Densities are then associated with each vertex and fluxes between boxes are associated with the edge connecting the two vertices, cf. Fig. 9. A drawback of this vertex-based scheme is the requirement of Delaunay meshes, which are very challenging to generate [69]. Rupp et al. proposed a cell-centered discretization scheme, where the cells (triangles, tetrahedra, etc.) are taken as boxes and hence the method is suitable for arbitrary meshes [70]. However, to account for the wide-spread use of vertex-based discretizations, we will consider a vertex-based discretization of the SHE equations in the following.

**Fig. 10:**
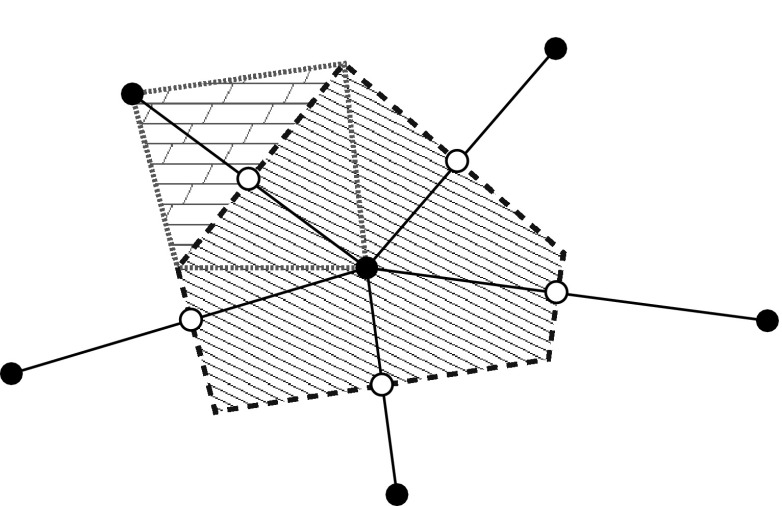
Unknowns expansion coefficients $g_{l,m}$ with even *l* are associated with vertices (*filled circles*), while those with odd *l* are associated with the dual boxes centered at an edge connecting two vertices (*open circles*)

Let $B_i$ denote the Voronoi box at vertex *i*, and $B_{i,j}$ the box associated with the dual box obtained from combining the contributions of the boxes $B_i$ and $B_j$ associated with the edge joining the vertices *i* and *j*, cf. Fig. 10. This results in a conforming decomposition of the simulation domain for structured and unstructured grids, i.e., both $\cup _i B_i$ and $\cup _{i,j; i < j} B_{i,j}$ exactly cover the simulation domain if the underlying mesh is sufficiently regular. For a discretization of both the even-order and odd-order equations, the velocity $\varvec{v}$ and the density of states *Z* (and thus also $\hat{\varvec{j}}_{l,m}^{l^\prime , m^\prime }$ and $\varvec{\varGamma }_{l,m}^{l^\prime , m^\prime }$) are assumed to be piecewise constant with respect to the spatial coordinate in each box $B_{i,j}$. Similarly, the unknown expansion coefficients $g_{l^\prime ,m^\prime }$ are assumed to be constant over their associated boxes $B_i$ and $B_{i,j}$ and energy interval $[H^-, H^+]$, respectively. Furthermore, we only need to focus our attention on the discretization of the free streaming operator, because the scattering operator does not contain any spatial derivatives and is thus not affected by the *H*-transformation [40, 41]. Integration of the free streaming operator in the even-order equations (23) over the box $B_i$ and the energy range $[H^-, H^+]$ leads to

36 $$\begin{aligned} \int _{H^-}^{H^+} \int _{B_i}&\nabla _{\varvec{x}} \cdot \hat{\varvec{j}}_{l,m}^{l^\prime , m^\prime } g_{l^\prime ,m^\prime } - \hat{T} \varvec{F} \cdot \varvec{\varGamma }_{l,m}^{l^\prime , m^\prime } g_{l^\prime ,m^\prime } \, \mathrm {d}\varvec{x} \, \mathrm {d}H . \end{aligned}$$

Gauss’ Theorem applied to the first term transforms the divergence into a normal derivative with respect to the surface $\partial B_i$. The resulting normal derivative in the surface integral is approximated by a finite difference approximation with respect to the edge of length $d_{i,j}$ connecting the box centers $\varvec{x}_i$ and $\varvec{x}_j$ of the boxes $B_i$ and $B_j$, respectively. Splitting the integration over $B_i$ into the individual contributions $B_i \cap B_{i,j}$ results in

37 $$\begin{aligned}&\sum _j \int _{\partial B_i \cap B_{i,j}} g_{l^\prime ,m^\prime } \varvec{n}_{i,j} \cdot \int _{H^-}^{H^+} \hat{\varvec{j}}_{l,m}^{l^\prime , m^\prime } \, \mathrm {d}H \, \mathrm {d}\varvec{x}\nonumber \\&\quad -\, \sum _j \int _{B_i \cap B_{i,j}} g_{l^\prime ,m^\prime } \hat{T}\varvec{F} \cdot \int _{H^-}^{H^+} \varvec{\varGamma }_{l,m}^{l^\prime ,m^\prime } \, \mathrm {d}H \, \mathrm {d}\varvec{x} , \end{aligned}$$

where $\varvec{n}_{i,j}$ is the outward-pointing unit normal vector of $B_i$ at the box interface $B_i \cap B_{i,j}$. The integrals over energy are independent of the unknowns and only enter as coefficients into the discrete system. With the volumes $V_{i,j} = \mathrm {vol}(B_i \cap B_{i,j})$ and the interface areas $A_{i,j} = \mathrm {vol}(\partial B_i \cap B_{i,j})$, we thus obtain the discrete form

38 $$\begin{aligned}&\sum _j A_{i,j} \int _{H^-}^{H^+} \hat{\varvec{j}}_{l,m}^{l^\prime , m^\prime } \, \mathrm {d}H \cdot \varvec{n}_{i,j} g_{l^\prime ,m^\prime }\nonumber \\&\quad -\, \sum _j V_{i,j} \hat{T} g_{l^\prime ,m^\prime } \varvec{F} \cdot \int _{H^-}^{H^+} \varvec{\varGamma }_{l,m}^{l^\prime , m^\prime } \, \mathrm {d}H . \end{aligned}$$

Because of the even-odd-coupling of the free streaming operator, all nonzero coefficients in (38) carry odd $l^\prime$, hence all $g_{l^\prime , m^\prime }$ are taken from the dual box $B_{i,j}$.

Integration of the streaming operator for the odd-order adjoint equations (24) over the adjoint box $B_{i,j}$ and the energy range $[H^-, H^+]$ results after an application of Gauss’ Theorem in

39 $$\begin{aligned}&\sum _{k \in \{i,j\}} \int _{\partial B_{i,j} \cap B_k} g_{l^\prime ,m^\prime } \varvec{n} \, \mathrm {d}\varvec{x} \cdot \int _{H^-}^{H^+} \hat{\varvec{j}}_{l,m}^{l^\prime , m^\prime } \, \mathrm {d}H\nonumber \\&\quad +\, \sum _{k \in \{i,j\}} \int _{B_{i,j} \cap B_k} g_{l^\prime ,m^\prime } \hat{T} \varvec{F} \, \mathrm {d}\varvec{x} \cdot \int _{H^-}^{H^+} \varvec{\varGamma }^{l,m}_{l^\prime , m^\prime } \, \mathrm {d}H . \end{aligned}$$

Since $g_{l^\prime ,m^\prime }$ was taken to be piecewise constant for the discretization, there holds

$$\begin{aligned}&\int _{\partial B_{i,j} \cap B_i} g_{l^\prime ,m^\prime } \varvec{n} \, \mathrm {d}\varvec{x} = A_{i,j} g_{l^\prime ,m^\prime } \varvec{n}_{j,i} ,\nonumber \\&\int _{\partial B_{i,j} \cap B_j} g_{l^\prime ,m^\prime } \varvec{n} \, \mathrm {d}\varvec{x} = A_{i,j} g_{l^\prime ,m^\prime } \varvec{n}_{i,j}\nonumber \end{aligned}$$

by using the path independence of the surface integral inside the boxes $B_i$ and $B_j$. The discrete form of the odd-order equations is thus obtained as

40 $$\begin{aligned}&A_{i,j} \int _{H^-}^{H^+} \hat{\varvec{j}}_{l,m}^{l^\prime , m^\prime } \, \mathrm {d}H \cdot \bigl ( \varvec{n}_{i,j} g_{l^\prime ,m^\prime }\vert _{B_j} + \varvec{n}_{j,i} g_{l^\prime ,m^\prime }\vert _{B_i} \bigr ) \, \mathrm {d}\varvec{x}\nonumber \\&\quad +\, \bigl ( V_{i,j} g_{l^\prime ,m^\prime } \vert _{B_i} + V_{j,i} g_{l^\prime ,m^\prime } \vert _{B_j} \bigr ) \hat{T} \varvec{F} \cdot \int _{H^-}^{H^+} \varvec{\varGamma }^{l,m}_{l^\prime , m^\prime } \, \mathrm {d}H . \end{aligned}$$

Due to the odd-to-even coupling property, only coefficients with even $l^\prime$ are nonzero, hence all $g_{l^\prime , m^\prime }$ are well defined on the boxes $B_i$ and $B_j$.

The discrete forms (38) and (40) can be used for structured as well as unstructured meshes on which a Voronoi-based finite volume scheme is possible. On structured grids, they exactly result in the discrete equations derived by Hong et al. [41] using a dimensional splitting.

### The role of spherical symmetry

The coupling between the projected equations for different spherical harmonics is primarily determined by the terms $\varvec{j}_{l,m}^{l^\prime , m^\prime }$, $\varvec{\varGamma }_{l,m}^{l^\prime , m^\prime }$, $Q_{\eta ; l,m}^{\mathrm {in}; l^\prime , m^\prime }$, and $Q_{\eta ; l,m}^{\mathrm {out}; l^\prime , m^\prime }$. For isotropic band structures, i.e., when there is no angular dependence of the velocity and the density of states, it is possible to factor the coupling terms (9) and (17) as

41 $$\begin{aligned}&\varvec{j}_{l,m}^{l^\prime , m^\prime } = v(\varvec{x}, H) \varvec{a}_{l,m}^{l^\prime , m^\prime } , \end{aligned}$$

42 $$\begin{aligned}&\varvec{\varGamma }_{l,m}^{l^\prime , m^\prime } = \varGamma (\varvec{x}, H) \varvec{b}_{l,m}^{l^\prime , m^\prime } , \end{aligned}$$

where $\varvec{a}_{l,m}^{l^\prime , m^\prime }$ and $\varvec{b}_{l,m}^{l^\prime , m^\prime }$ are independent of energy and can thus be precomputed. As shown by Rupp et al. $\varvec{a}_{l,m}^{l^\prime , m^\prime }$ and $\varvec{b}_{l,m}^{l^\prime , m^\prime }$ exhibit an interesting coupling structure: For given *l* and *m*, both coupling terms can take nonzero values only if $l^\prime = l \pm 1$ and $m^\prime = \pm \vert m \vert \pm 1$. This greatly simplifies the coupling structure for isotropic compared to anisotropic band structures.

Spherical symmetry of the band structure also has a tremendous impact on the numerical complexity induced by the scattering operators. For a single scattering process identified by $\eta$, the contribution to the total scattering rate *s* in (29) after a transformation to $(\varepsilon , \theta , \varphi )$ coordinates in general is

$$\begin{aligned} s_{\eta } = s_{\eta }(\varvec{x}, \varepsilon , \theta , \varphi , \varepsilon ^\prime , \theta ^\prime , \varphi ^\prime ) \end{aligned}$$

and thus depends on the angles. Consequently, $s_{\eta }$ needs to be considered in all spherical projections and may thus result in an complicated coupling among the individual expansion coefficients $g_{l,m}$. However, if the transition rate $s_{\eta }$ is approximated as velocity randomizing [3, 49], it only depends on the energies. Therefore, the angular terms can be integrated directly to obtain

43 $$\begin{aligned} s_{\eta } \sim \delta _{l,l^\prime } \delta _{m,m^\prime } , \end{aligned}$$

where $\delta _{i,j}$ is zero whenever $i \ne j$ and unity otherwise. In such a case, the scattering operator does not couple different expansion coefficients $g_{l,m}$ at all, hence the coupling among expansion coefficients with different index pairs (*l*, *m*) and $(l^\prime , m^\prime )$ is solely determined by the free streaming operator.

**Fig. 11:**
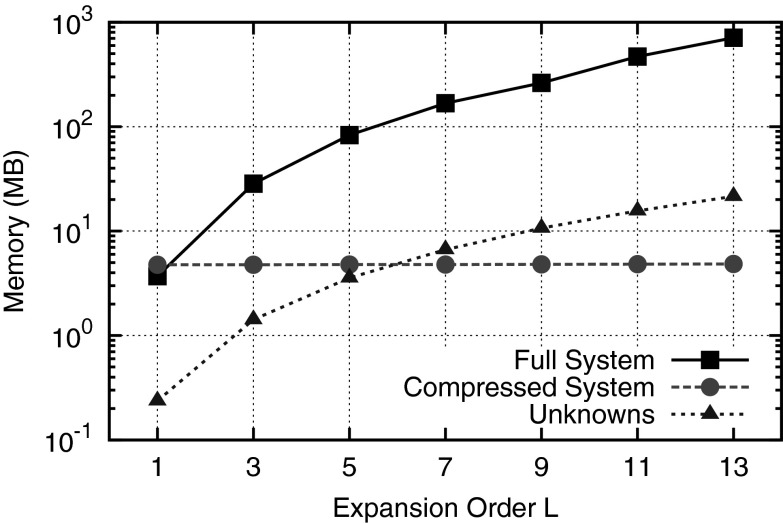
Exemplary comparison of the memory requirements of the full system and the compressed system when using the Kronecker product structure of the equations with the memory needed for the storage of the system unknowns only

The decouplings (41), (42), and (43) also induce a Kronecker product structure of the system matrix. Kronecker product structures are common for spectral methods [71] and can be exploited to reduce the memory requirements of the system matrix. Rupp et al. have shown that such a Kronecker product structure allows for a compressed storage of the full system matrix for the SHE method, resulting in the following two advantages [72]: First, the memory-efficient representation based on Kronecker products enables higher cache utilization, reduces data transfers from global random access memory, and thus increases performance. Second, one can quickly change the expansion order for a given electrostatic potential without traversing the mesh, but by only adjusting the SHE coupling matrices. However, significant memory savings can only be seen beyond first order, cf. Fig. 11. Furthermore, good preconditioners usually require the reconstruction of the full system matrix, in which case the memory savings are lost. A possible way to obtain good preconditioners without explicitly storing the full system matrix for higher-order SHE is to compute the preconditioner for a low-order SHE system and use this preconditioner to solve the high-order system. Such a strategy was successfully used by e.g., Brown for the solution of the Stokes problem using nodal finite elements [73].

**Fig. 12:**
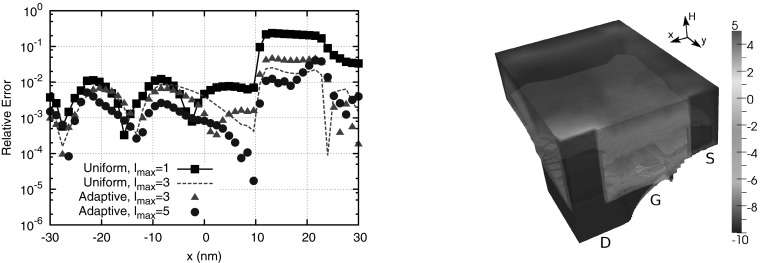
*Left* Comparison of the relative error of the average carrier energy along a straight line from source to drain in a MOSFET. *Right* Plot of the error indicator within the nMOSFET in $(\varvec{x}, H)$-space. The error indicator is particularly high in the channel and at the high-energy tail of the distribution function in the drain

### Self-consistency

So far we have only considered the BTE for a given electrostatic potential $\psi$. An approximation to the electrostatic potential may, for instance, be obtained from a solution of the drift-diffusion model. Certain insights can be obtained in such a setting, most notably the overall shape of the distribution function at higher energies. However, only a fully self-consistent solution of the Poisson equation

44 $$\begin{aligned} \varDelta \psi = |q| (n - p + \mathcal {C}) \end{aligned}$$

with electron density *n*, hole density *p*, and net doping $\mathcal {C}$ together with the BTE provides accurate values for quantities such as the current density.

Similar to moment-based methods, two main methods are in use for obtaining self-consistency. The first method is the *Gummel method* [74]. It relies on an iterated solution of the Poisson equation and the drift-diffusion equation or the BTE for each carrier type. For unipolar devices, the second carrier type may also be ignored. The distribution function obtained from a solution of the BTE is translated into a carrier density via the relation

45 $$\begin{aligned} n = \frac{1}{Y^{0,0}} \int _0^\infty g_{0,0} \, \mathrm {d}\varepsilon \end{aligned}$$

for electrons and similarly for holes. An additional damping is commonly applied in analogy to moment-based methods to improve robustness of the iteration. Furthermore, the electrostatic potential obtained from solutions of moment-based models can be used as an initial guess for the Poisson-BTE system.

The second method for achieving self-consistency is Newton’s method, through which quadratic convergence close to the solution is obtained. Newton’s method requires the solution of a system described by the full Jacobi matrix of the coupled equations in each step. While partial derivatives of the densities with respect to the SHE coefficients are easily obtained from (45), additional care needs to be taken when computing the partial derivatives of the terms in the BTE with respect to the potential $\psi$. In a formulation based on kinetic energy, only the terms involving the force $\varvec{F}$ lead to additional contributions to the Jacobi matrix. On the other hand, a formulation based on total energy *H* needs to account for the dependence of the total energy *H* on the potential. In particular, $\varvec{j}_{l,m}^{l^\prime , m^\prime }$ and $\varvec{\varGamma }_{l,m}^{l^\prime , m^\prime }$ depend on $\psi$ through the total energy *H*.

Since Newton’s method may fail with a poor initial guess, the initial nonlinear iterations are often carried out using Gummel’s method. When the current iterate is closer to the actual solution, Newton’s method is then used, ultimately resulting in quadratic convergence. In certain scenarios, the SHE method may also exhibit higher numerical stability than moment-based methods: Jungemann et al. reported superior numerical stability of the SHE method during their study of impact ionization effects [75].

### Adaptive variable-order scheme

The number of SHE coefficients in (13) is $(l_{\max } + 1)^2$. Contrary to moment-based methods, a SHE truncated after the zeroth-order term still represents the equilibrium solution exactly. Moreover, for the non-equilibrium case, it is known that SHE coefficients decay rapidly according to the smoothness of the underlying function.

**Fig. 13:**
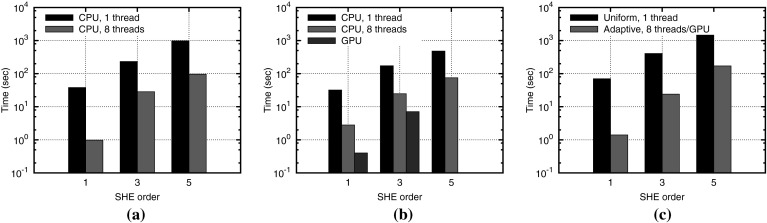
Comparison of preconditioner setup time (**a**), iterative solver time (**b**), and total solver time (**c**) for the SHE method for a given electrostatic potential [77]

From a computational standpoint, it is desirable to use a small maximum expansion order $l_{\max }$ to minimize the numerical complexity. On the other hand, first-order expansions provide, despite their appealing properties discussed in Sect. 2, insufficient accuracy for scaled-down devices under quasi-ballistic transport conditions. Certain regions of a device, for example deep in the bulk, do not provide any significant contributions to carrier transport, hence the additional computational effort for high-order expansions may not be necessary. Similarly, a high-order expansion may not be necessary at high energies where the distribution function takes very small values.

Rupp et al. developed a variable-order scheme to select appropriate expansion orders across the device [76]. Their scheme allows for the specification of the maximum expansion order depending on the location in $(\varvec{x}, H)$-space, i.e., $l_{\max } = l_{\max }(\varvec{x}, H)$. Alternatively, the scheme may also be interpreted as selecting $l_{\max }$ fixed throughout the whole simulation domain, but certain expansion coefficients are a-priori set to zero because they are expected (or known) to be insignificant.

Since the manual specification of expansion orders is impractical for engineering purposes, Rupp et al. also proposed adaptive schemes for automatically selecting the expansion order in a bootstrap procedure [76]: Starting from a first-order SHE, the expansion order is increased to third order in regions where the SHE truncation error is large (Fig. 12). Three schemes have been proposed for the detection of these regions: One is based on the relative weights for the computation of a target quantity such as the current density, the second monitors the decay of the expansion orders with respect to *l* for fixed $(\varvec{x}, H)$, and the third is a residual-based scheme similar to those typically used with finite element methods. After the expansion order is locally increased, another solution of the Boltzmann-Poisson system is computed and the adaption procedure repeated until convergence.

An adaptive variable-order scheme is particularly beneficial when used with structured grids in two or three spatial dimensions. The reason is that the tensor construction of structured grids enforces that high resolutions in one part of the device also result in high resolution in other, possibly less important, parts of the device. The adaptive variable-order scheme will then select a low expansion order in these less important parts of the device. Conversely, if the savings in computational cost for unstructured grids instead of structured grids are already high, the additional savings from an adaptive variable-order scheme are smaller [77].

### Parallelization

Iterative methods are preferred over direct methods for the solution of large systems of linear equations such as those obtained in each nonlinear iteration step when using the SHE method. At the same time, the use of iterative methods typically requires good preconditioners to accelerate the convergence process. Jungemann et al. reported successful convergence using preconditioners based on incomplete LU factorizations for a formulation based on kinetic energy [40]. Vecchi et al. observed a decoupling of the *H*-transformed SHE equations into several subsystems depending on the inelastic scattering mechanisms employed and on the grid spacing in energy direction [25]. Rupp et al. extended these ideas to a general block preconditioning scheme, where the preconditioner can be built and applied in parallel for each discrete total energy [78]. The approach is based on the observation that scaled-down devices are increasingly dominated by quasi-ballistic transport. Therefore, the action of the full system matrix is captured in good approximation by a system matrix without inelastic scattering events. In the absence of inelastic scattering, the system matrix decouples into independent subsystems for each discrete total energy. Therefore, Rupp et al. proposed to build a parallel block-preconditioner from a system without inelastic scattering events in order to solve the full system including inelastic scattering. Since the number of discrete energies is in the hundreds, enough parallelism is available even for massively parallel architectures such as GPUs. Performance gains of up to an order of magnitude over a single-threaded implementation were reported on a shared memory system, cf. Fig. 13 [78]. These gains partly stem from the smaller computational effort in computing the preconditioner due to the absence of inelastic scattering, and partly from a better utilization of the underlying hardware.

In principle, the block preconditioning scheme can also be used on smaller-sized clusters. For large-scale simulations, preconditioners based on incomplete LU factorizations are known to scale rather poorly. Hence, better parallel preconditioners, particularly multigrid preconditioners, are desirable, but have not been investigated for the SHE method yet.

## Selected applications

In this section, we summarize selected application areas for which the SHE method has been employed successfully. We focus on applications where the SHE method provides significant progress over other methods used in the past. Thus, our discussion is not meant to be an exhaustive list of possible application areas for the SHE method.

**Fig. 14:**
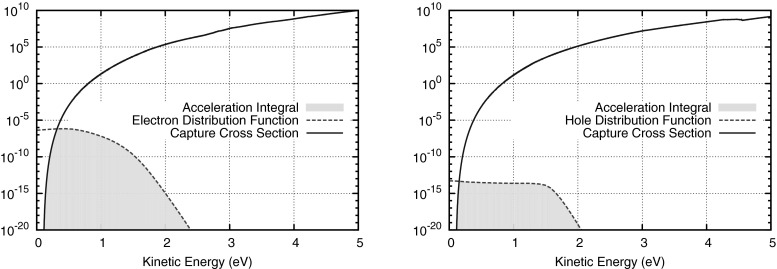
Exemplary distribution functions and acceleration integrals (*shaded area*) for electrons (*left*) and holes (*right*) in the middle of an artificial short-channel (25 nm) *n*-channel MOSFET. The high-energy tail of the distribution function needs to be resolved accurately such that after multiplication by the collision cross section $S(\varepsilon )$ the acceleration integral (46) can be computed reliably

### Noise

The ongoing interest in further improving the noise performance of semiconductor devices is hampered by the inability of the Monte Carlo method to simulate the noise behavior of devices at technically relevant frequencies in the lower GHz range [75]. Jungemann has demonstrated that the SHE method is well suited for the simulation of noise by solving the Langevin-Boltzmann equation in the frequency domain [79, 80]. The deterministic nature of the SHE method also allows for the accurate simulation of rare events and slow processes, which for example occur in the case of deep traps. For these reasons, Dinh et al. used the SHE method as a reference to benchmark a commercial noise solver based on the drift-diffusion and hydrodynamic models [81].

### Small-signal analysis

Small-signal analysis requires that the BTE is linearized together with the Poisson equation at the bias point under inspection, hence fluctuations of the electrostatic potential play a role [46]. Such fluctuations can be considered directly through the force term when using a formulation based on kinetic energy $\varepsilon$ as in (18), but additional attention is required when using the *H*-transformation. Since the location of the band edge in $(\varvec{x}, H$)-space depends on the electrostatic potential, a naive application of the SHE method for small-signal analysis yields time-varying coefficients. Lin  et al. proposed to fix the stationary part of the electrostatic potential and to keep an additional derivative with respect to total energy for the linearization [82]. This resolves the problems with time-varying coefficients, but results in an additional coupling of adjacent discrete energies.

### Hot carrier degradation

High electric fields, as they are common in the pinch-off region of a MOSFET, lead to a strong acceleration of carriers. A few carriers may reach energies up to several electron volts, which is sufficient for creating electron–hole pairs or for surpassing the oxide energy barrier. These so-called *hot carriers* are of utmost interest for the study of device degradation phenomena [83, 84].

For the modeling of hot-carrier effects such as impact ionization or interface state generation, the collision cross section $S(\varepsilon )$ typically grows quickly above a threshold energy $\varepsilon ^{\mathrm {th}}$. The total rate $G(\varvec{x}, t)$ is obtained from the acceleration integral

46 $$\begin{aligned} G(\varvec{x}, t) \sim \int _{\varepsilon ^{\mathrm {th}}}^\infty f(\varvec{x}, \varepsilon , t) S(\varepsilon ) \, \mathrm {d}\varepsilon \end{aligned}$$

and relies on a good resolution of the distribution function at higher energies, cf. Fig. 14, for which carrier-carrier scattering and impact ionization are essential [85, 86].

The need for a high resolution of high-energy tails has long- hampered scientific progress because of excessive execution times obtained with the Monte Carlo method. First results for a long-channel MOSFET were reported only recently [87]. As a remedy, simplified versions of the model are used in practice [83, 88]. With the availability of the SHE method, these simplifications are no longer necessary.

### Avalanche breakdown

The abrupt onset and strong nonlinear behavior makes the simulation of avalanche breakdown during the switching of power devices numerically very challenging. Also, the breakdown is not immediate: At typical breakdown voltages of several tens of Volts, the breakdown may need hundreds of picoseconds to fully develop. With a time step restriction of a femtosecond or less, the Monte Carlo method is therefore not suitable for the simulation of avalanche breakdown.

Jabs et al. developed a continuation method to deal with these challenges and presented simulation results using the SHE method for the avalanche breakdown of a 2D vertical power MOSFET and a *pn*-diode at a reverse biases of up to 39 Volt [58, 89]. They introduced a penalty parameter through which they controlled the current and avoided divergence in the numerical solver. Also, to address the ill-conditioning of the full system matrix, they introduced a splitting of the system matrix into a contribution from the BTE without impact ionization (matrix $\varvec{B}$) and a contribution from impact ionization (matrix $\varvec{Q}$). By using $\varvec{B}$ as a preconditioner for a Richardson iteration to solve the full system described by the matrix $\varvec{B} - \varvec{Q}$, they obtained a robust numerical scheme.

## Outlook

In the following, we discuss possible future enhancements and applications of the SHE method. Based on our own experience, we consider an extension of the SHE method to more materials, the possibility to run large-scale simulations, and the solution of the transient BTE using the SHE method to be the most promising topics for future exploration.

### More materials

The use of the SHE method for semiconductor device simulations has been focused on silicon and silicon-germanium devices. An exception to this observation is reported by Ramonas and Jungemann, who investigated the electron–phonon interaction in gallium-nitride high-electron-mobility transistors using the SHE method [90, 91]. Kargar et al. reported the use of the SHE method coupled with the Poisson and Schrödinger equations for gallium arsenide [92]. Extensions to other popular materials or material combinations such as silicon-carbide will increase the overall attractiveness and versatility of the SHE method.

### Large-scale simulations

The additional energy coordinate implies that the SHE method requires about two to three orders of magnitude more memory than macroscopic models such as the drift-diffusion model. Today’s machines with tens of Gigabytes of main memory provide enough resources to run spatially one- and two-dimensional device simulation using the SHE method. Even fully three-dimensional device simulations are possible, yet only at moderate resolution and without carrier-carrier scattering [77]. Consequently, there is clear benefit of employing the SHE method on distributed memory machines, including supercomputers. The added benefit of such large-scale simulations is that devices can not only be simulated at higher accuracy, but for a given resolution one can also obtain shorter execution times through the use of more cores and memory channels. This is particularly interesting in an engineering environment, where short turnaround times are of importance.

### SHE for the transient BTE

The SHE method has so far been employed for the stationary BTE only, yet a solution of the transient case would allow for a study of the long-time behavior in devices at an unprecedented level of detail. While solvers for the transient BTE are readily available in other application areas such as the simulation of rarefied gas flows, the BTE for semiconductors does not allow for a direct application of the techniques in these other areas. The primary reason is that the external force term in the BTE vanishes in other application areas. Therefore, numerical instabilities are less a concern there, as there is no *H*-transformation required and thus no dependence of the simulation domain on an external potential is encountered.

The application of a time discretization to the SHE equations using the *H*-transformation requires the transfer of the current solution at time step *k* to the next time step $k+1$. Since in general the electrostatic potential changes from time step *k* to time step $k+1$, an interpolation of the current solution is necessary due to the shift of the band edge. This interpolation, however, results in interpolation errors, which ultimately prevent charge conservation. It is not yet clear whether and how these issues can be addressed. A possible path forward is to relax or even drop the *H*-transformation and work with discretizations based on kinetic energy.

## Summary

The SHE method has reached a level of maturity where it is not only an attractive alternative to the established Monte Carlo method, but at the same time allows for conducting research on phenomena which cannot be simulated with a stochastic method. The absence of stochastic fluctuations enables simulations of noise and exact small- signal analysis at an unprecedented accuracy and for a much larger range than ever before. While the high dimensionality of the BTE implies that the SHE method is still very demanding in terms of memory consumption, the method is considerably less costly when compared to other direct approaches.

A drawback of the SHE method for wide-spread adoption is the fact that the method is fairly complex in terms of the mathematics involved. The development of a SHE solver from scratch easily takes weeks or months of concentrated effort. However, the availability of a free open source simulator (ViennaSHE^1^) lowers the entry barrier considerably. Also, commercial implementations are available which enable the use of the SHE method without any coding effort at all.

While the SHE method provides more insight than moment-based methods, it is unlikely that the SHE method will ever fully replace moment-based methods. Instead, the SHE method provides another method in a full hierarchy of different solution approaches. For a given application it is thus advisable to select the fastest method which fulfills the requirements on accuracy. If the particular application requires the fast computation of carrier distribution functions in one way or another, the SHE method is likely to be the best choice.

## Footnotes

1 http://viennashe.sourceforge.net/
